# Independent and cumulative coeliac disease-susceptibility loci are associated with distinct disease phenotypes

**DOI:** 10.1038/s10038-020-00888-5

**Published:** 2021-01-15

**Authors:** Juliana X. M. Cerqueira, Päivi Saavalainen, Kalle Kurppa, Pilvi Laurikka, Heini Huhtala, Matti Nykter, Lotta L. E. Koskinen, Dawit A. Yohannes, Elina Kilpeläinen, Anastasia Shcherban, Aarno Palotie, Katri Kaukinen, Katri Lindfors

**Affiliations:** 1 Coeliac Disease Research Center, Faculty of Medicine and Health Technology, Tampere University, Tampere, Finland; 2 Research Programs Unit, Immunobiology, and the Haartman Institute, Department of Molecular Genetics, University of Helsinki, Helsinki, Finland; 3 Center for Child Health Research, Tampere University and Tampere University Hospital, Tampere, and the University Consortium of Seinäjoki, Seinäjoki, Finland; 4 Faculty of Social Sciences, Tampere University, Tampere, Finland; 5 Laboratory of Computational Biology, Faculty of Medicine and Health Technology, Tampere University, Tampere, Finland; 6 Institute for Molecular Medicine Finland (FIMM), University of Helsinki, Helsinki, Finland; 7 Broad Institute of the Massachusetts Institute of Technology and Harvard University, Cambridge, MA USA; 8 Psychiatric & Neurodevelopmental Genetics Unit, Department of Psychiatry, Analytic and Translational Genetics Unit, Department of Medicine, and the Department of Neurology, Massachusetts General Hospital, Boston, MA USA; 9 Department of Internal Medicine, Tampere University Hospital, Tampere, Finland

**Keywords:** Coeliac disease, Genome-wide association studies, Genetic predisposition to disease, Genetic databases, Disease genetics

## Abstract

The phenotype of coeliac disease varies considerably for incompletely understood reasons. We investigated whether established coeliac disease susceptibility variants (SNPs) are individually or cumulatively associated with distinct phenotypes. We also tested whether a polygenic risk score (PRS) based on genome-wide associated (GWA) data could explain the phenotypic variation. The phenotypic association of 39 non-HLA coeliac disease SNPs was tested in 625 thoroughly phenotyped coeliac disease patients and 1817 controls. To assess their cumulative effects a weighted genetic risk score (wGRS39) was built, and stratified by tertiles. In our PRS model in cases, we took the summary statistics from the largest GWA study in coeliac disease and tested their association at eight *P* value thresholds (*P*_T_) with phenotypes. Altogether ten SNPs were associated with distinct phenotypes after correction for multiple testing (*P*_EMP2_ ≤ 0.05). The *TLR7/TLR8* locus was associated with disease onset before and the *SH2B3/ATXN2*, *ITGA4/UBE2E3* and *IL2/IL21* loci after 7 years of age. The latter three loci were associated with a more severe small bowel mucosal damage and *SH2B3/ATXN2* with type 1 diabetes. Patients at the highest wGRS39 tertiles had OR > 1.62 for having coeliac disease-related symptoms during childhood, a more severe small bowel mucosal damage, malabsorption and anaemia. PRS was associated only with dermatitis herpetiformis (*P*_T_ = 0.2, *P*_EMP2_ = 0.02). Independent coeliac disease-susceptibility loci are associated with distinct phenotypes, suggesting that genetic factors play a role in determining the disease presentation. Moreover, the increased number of coeliac disease susceptibility SNPs might predispose to a more severe disease course.

## Introduction

Food antigens do not generally cause a systemic immune response in healthy individuals, but rather lead to the induction of oral tolerance. However, in approximately 1–2% of individuals, the ingestion of dietary gluten results in the development of coeliac disease, an immune-mediated enteropathy [1]. In some patients, coeliac disease develops already in early childhood, while in others, the tolerance to gluten is lost at a considerably older age. A hallmark of the disease is gluten-dependent small bowel mucosal damage that ranges from minor inflammatory changes to total villous atrophy with crypt hyperplasia [2]. The enteropathy is often coupled with malabsorption and gastrointestinal symptoms, such as diarrhoea, but the disease also presents with diverse extraintestinal manifestations affecting various organs including skin and musculoskeletal system [2, 3]. This multifaceted clinical picture is further diversified by several associated conditions, such as type 1 diabetes and autoimmune thyroidal disease [2].

The predisposition for coeliac disease runs in families, and relatives of coeliac patients are at an increased risk [4]. The disease susceptibility is largely conferred by human leucocyte antigen (HLA) haplotypes encoding for DQ2 or DQ8 heterodimers, which are necessary but not sufficient for disease development [2]. Genome-wide association (GWA) and follow-up studies have identified 94 SNPs in 43 non-HLA risk loci that by themselves modify the disease risk modestly [5–8]. However, combining their independent cumulative effects into a genetic risk score (GRS) improves the prediction of the disease risk [9]. Moreover, by combining the loci of small effect, including those that do not achieve genome wide significance into a polygenic risk score (PRS), it has been possible to examine the influence of several thousands of risk alleles to coeliac disease susceptibility [7, 10].

Currently, there’s only limited information about the connection of the non-HLA risk SNPs with different coeliac disease phenotypes. Therefore, the aim of this study was to investigate whether previously identified coeliac disease-susceptibility SNPs are associated with distinct disease phenotypes and to gain insight into possible biological pathways and processes underlying the identified genotype–phenotype associations. Moreover, the objective was to study whether GRS or PRS give clues to the factors contributing to the clinical heterogeneity of the disease.

## Materials and methods

### Study population

Altogether 1048 biopsy-proven coeliac disease patients were recruited by a nationwide newspaper advertisements and the assistance of the Finnish Coeliac Society in Tampere University and Tampere University Hospital 2005–2010. The study protocol was approved by the Regional Ethics Committee of Tampere University Hospital and all study subjects or young children’s legal guardians gave written informed consent. All participants were interviewed either by a physician or by a study nurse with expertise in coeliac disease. The structured interviews included questions on coeliac disease diagnosis, symptoms at the time of diagnosis and in childhood, and associated medical conditions. All relevant medical information was confirmed from the patient records. Whole blood samples were drawn for genetic analysis. As the presence of relatives can lead to genetic bias (inflation of type 1 error), the current study considered only one coeliac case per family resulting in 625 cases grouped into relevant phenotypes (Table 1). The median age of the patients was 41 ranging from 0.5 to 79 years. The individual was selected randomly among the family members with full genotype data available. The healthy controls (*n* = 1817) with information on gender and HLA-type were obtained from the FINRISK and Health 2000 population cohorts [11]. Ethical committee’s approvals were available from the National Public Health Institute’s ethical committee and the Ethical committee in epidemiology and public health at the hospital district of Helsinki and Uusimaa.

**Table 1:** Demographic data, clinical characteristics and selected coeliac disease-associated findings in 625 coeliac disease patients at diagnosis

	*n*	%
Female	489	78
Childhood diagnosis ≤7 years old	44	7
Coeliac disease-related symptoms during childhood	273	44
Gastrointestinal symptoms^a^	526	84
Malabsorption^b^	267	43
*Anaemia*	157	25
Extraintestinal manifestations^c^	263	42
*Dermatitis herpetiformis*	69	11
*Neurological disorders*	38	6
*Fractures*	70	11
Asymptomatic	53	8
Coeliac disease-associated conditions^d^	123	20
*Autoimmune thyroidal diseases*	100	16
*Type I diabetes*	18	3
Small bowel mucosal damage^e^		
*Total or subtotal villous atrophy*	361	66
*Partial villous atrophy*	185	34
HLA risk^f^		
* Intermediate/low*	527	84
*High*	98	16
Coeliac disease autoantibodies^g^		
*Positive*	279	94
*Negative*	19	6

^a^Diarrhoea, abdominal pain, flatulence, heartburn, nausea, vomiting

^b^Anaemia, vitamin and micronutrients deficiencies

^c^Dermatitis herpetiformis, rash, neurological and psychiatric disorders, fracture, arthralgia, tiredness, fatigue, osteoporosis, osteomalacia, osteopenia, aphthae, alopecia, growth retardation, elevated liver enzymes, dental defects, infertility

^d^Small bowel mucosal morphology available from 546 patients

^e^High risk (DQ2.5/DQ2.5; DQ2.5/DQ2.2), intermediate risk (DQ2.5/X, DQ2.2/D2.2, DQ2.2/X, DQ8/DQ8, DQ8/X), low risk (DQ7/X, DQ7/DQ7)

^f^autoantibodies at diagnosis available from 298 patients

### Genotyping and quality control

HLA-DQ typing was performed with the TaqMan chemistry, SSP DQB1 and DRB1 low-resolution kit (Olerup SSP AB, Saltsjöbaden, Sweden), or DELFIA^®^ Coeliac Disease Hybridization Assay Kit (PerkinElmer Life and Analytical Sciences, Wallac Oy, Turku, Finland) [12, 13]. For patients and controls missing HLA-typing results the missing GWAS SNPs were further imputed in full HLA haplotypes with HIBAG R package [14].

All participants had been genotyped for a SNP set on an Illumina 610-Quad BeadChip array (Illumina Inc., San Diego, CA, USA) [5]. The SNPs with established risk for coeliac disease were selected. We included the 39 non-HLA coeliac disease SNPs identified in the earlier GWAS [5] and directly genotyped in our samples. As most of the SNPs identified in Trynka et al. study [6] were either not available at our array or had low genotyping frequency, they were imputed (further details under the “imputation” section).

Genotypes were stored in BC│Genome v.4.0 (BC│Platforms, Espoo, Finland) and quality checks performed [15]. The genotyped and imputed SNPs passed the quality control (QC) filters for missing genotype rate < 5%, missing genotype rates differences between the cases and controls (<3%), and minor allele frequency (MAF > 1%) [16]. All markers were (*P* > 1 × 10^−6^) in HWE in the controls [6, 16]. Allelic associations with phenotypes were tested in a case–control analysis and within cases, further detailed under the ‘statistical analysis’ section.

### Imputation

The genotypes of 94 coeliac disease  risk variants [5, 6] were selected to be phased and imputed by using a Finnish population-specific panel of 3775 high-coverage (25–30×) whole-genome sequences (SISu v3) as here described: dx.doi.org/10.17504/protocols.io.nmndc5e. SISu v3 panel was generated at the Broad Institute of MIT and Harvard and at the McDonnell Genome Institute at Washington University; and jointly processed at the Broad Institute.

Genotyping data produced with our chip platform were lifted over to genome build version 38 (GRCh38/hg38) following the protocol described here: 10.17504/protocols.io.nqtddwn. In sample-wise quality control (QC), samples with sex discrepancies, high genotype missingness (>5%), excess heterozygosity (±4SD) and non-Finnish ancestry were removed. In variant-wise QC, variants with high missingness (>2%), deviation from Hardy–Weinberg equilibrium (HWE) (*P* < 1e-6) and minor allele count <3 were removed. Pre-phasing (default parameters, except the number of conditioning haplotypes was set to 20,000) and phasing of genotyped data were performed with Eagle 2.3.5 (https://data.broadinstitute.org/alkesgroup/Eagle/)

Imputation was carried out with Beagle 4.1 (version 08Jun17.d8b, https://faculty.washington.edu/browning/beagle/b4_1.html) as described in the following protocol: dx.doi.org/10.17504/protocols.io.nmndc5e. Variant callset was produced by following GATK best-practices. Genotype-, sample- and variant-wise QC was applied in an iterative manner by using the Hail framework v0.1 [https://github.com/hail-is/hail].

In the post-imputation QC, the SNPs with good imputation quality metrics (INFO score ≥0.8) and MAF > 0.01 were included. The VCF files with the genotypes probabilities dosages were handled by using bcftools (https://samtools.github.io/bcftools/)

### Functional identification of enriched pathways related to the phenotype-associated SNPs

Given that coeliac disease associated SNPs might not be causal variants but situated in their close proximity e.g in high linkage disequilibrium (LD) with them, FUMA (Functional Mapping and Annotation of GWAS) platform [17] was used for the functional annotation of the 39 genotyped SNPs. Publicly available GWAS summary statistic results in which our cohort has been included [5] and a pre-defined list of associated SNPs with phenotypes in this study were provided. All proxies in LD (*r*^2^ ≥ 0.8) with the phenotype-associated SNPs were identified using HaploReg v.4.1 [18]. The RegulomeDB 2.0 [19] was used to assign all the variants a score ranging from 1a to 7. Scores from 1a to 3a are likely to affect the expression of a gene. The lesser the score the higher likehood, thus 1a indicating a maximum and 3a a minimum likelihood to affect gene expression [19]. Variants scoring ≥3a but predicted as likely deleterious [Combined Annotation Dependent Depletion (CADD) score closest or >12.37)] were also included [20]. For those phenotype-associated SNPs scoring >3a, we selected their proxy with the lowest RegulomeDB score [19]. Using publicly available databases to study tissue-specific gene expression, we examined significant eQTLs associations (FDR < 0.05) with the functional variants, located nearby (cis-eQTL) or distal (trans-eQTL) to genes. They were assessed in the whole and peripheral blood using the Blood eQTL [21], BIOS QTL [22] and eQTLGen [23] browsers. Moreover, as the phenotype of coeliac disease varies considerable and the symptoms may affect the function of several cell types tissues we also used the Genotype-Tissue Expression data (brain, nerve, colon, oesophagus, EBV-transformed lymphocytes, muscle-skeletal, pancreas, skin, small intestine, stomach, cultured fibroblasts, thyroid, whole blood) [24]. Their participation in pathways and biological processes were identified by using KEGG (*Kyoto Encyclopedia of Genes and Genomes*) and GO (*Gene Ontology*) databases [25].

### Genetic risk score

In order to assess the cumulative effects of the genotyped 39 non-HLA SNPs on phenotypes, we created a weighted GRS model (wGRS39), calculated by multiplying each allele by the natural logarithms of the previously reported OR values (β-coefficients) [5], followed by dividing their sum by the total number of alleles [9]. We analysed the wGRS39 model in tertiles, adjusted for sex, calculated according to the distribution of the average weighted risk alleles in the controls as published [26]. Based on these tertiles, all participants were categorised into the low-, medium- and high-risk groups.

### Polygenic risk score

We derived a PRS using summary statistics from the most recent and largest GWAS in coeliac disease with 24,269 participants [6] as the discovery dataset and tested its association with phenotypes. After extracting only cases in our cohort, we performed LiftOver (https://genome.ucsc.edu/cgi-bin/hgLiftOver) to update our genotype data to a recent human genome build (hg19) and were left with 624 cases and 526,118 SNPs. We applied quality filters as described above and constructed the PRS accordingly [27] using the ‘fastscore’ option in PRSice software package [28]. PRSs were calculated as the sum of all risk variants carried by that individual weighted by the *β*-coefficients reported in the discovery GWAS [6]. To account for independent association signals, clumping retained the SNP with smallest *P* value in each 250 kilobases window and removed all those in LD (*r*^2^ > 0.2) with this SNP [7]. SNPs in the MHC region with long range LD (29.7–33.3 megabases) were excluded [10]. PRS was calculated on a priori set of significant thresholds (*P* < 0.001, *P* < 0.01, *P* < 0.05, *P* < 0.1, *P* < 0.2, *P* < 0.3, *P* < 0.4, *P* < 0.5) to identify the best fit PRS that was most predictive of an association.

### Statistical analysis

Allelic associations using the genotyped 39 SNPs were tested by using the chi-squared method with one degree of freedom, measured by OR values and 95% confidence intervals. Logistic regression adjusted for SNP-gender interaction. We performed 10^4^ permutation tests, generating an empirical *P*_EMP2_ for the evaluation of subgroups with a small sample size and adjusting for multiple tests [16]. Associations were statistically significant at *P*_EMP2_ ≤ 0.05. The Breslow–Day (BD) test examined the homogeneity of the association’s OR [*P*_BD_ values < 0.02 (0.05/2)] in each male and gender strata [29].

Phenotype-association testing by using post-imputation genotype probabilities was performed under an additive genetic effect model using the frequentist likelihood score method implemented in SNPTEST v2.5.2 [30]. All the associations were adjusted for gender with the SNPTEST method Newml. For ChrX region we additionally used the ChrX-specific SNPTEST method with stratify-on gender option. Associations that reached out our permutation threshold (*P* value ≤ 0.001) were considered as statistically significant.

Using the Genetic Power Calculator [31], we computed the statistical power reached by the sample size set for each associated phenotype to detect allele association at permutation (*P* ≤ 0.001) and at nominal (*P* ≤ 0.01 and *P* ≤ 0.05) thresholds (detailed results in Supplementary Table S1). SNPs were assumed to be independent causal variants with no other causal variants in linkage disequilibrium (*D*′ = 1).

The eQTL associations by the selected variants located nearby (*cis*-eQTL) or distal (*trans*-eQTL) to genes were significant at a false discovery rate (FDR) < 0.05. An overall representation analysis adjusted by Benjamini & Hochberg procedure was performed by using WEB-based GEne SeT AnaLysis online toolkit [25] to identify significant (FDR < 0.05) eQTL gene-sets enriched in KEGG pathways and GO biological processes.

Logistic regression was used to test the associations of the wGRS39 tertiles with the coeliac phenotypes after comparing each group (medium [2nd tertile] and high [3rd tertile] wGRS39 risk categories) to a reference (low wGRS39 risk category, 1st tertile). The associations were quantified by ORs with 95% CIs, significant at *P* value < 0.05. In our case only model, we tested the associations between PRS and phenotypes using linear logistic regression, adjusting for age and sex. Multiple comparisons were addressed by applying 10^4^ permutations to identify the best fit PRS (*P*_EMP2_ ≤ 0.05). Results are presented as change in variance (*R*^2^), with both unadjusted *P* values and *P*_EMP2_ values.

All data analysis, conversion of required input formats in the imputation analysis and graphs were performed using PLINK 1.90 (https://www.cog-genomics.org/plink/1.9), PLINK 2.0 (https://www.cog-genomics.org/plink/2.0/), PRSice v2 and RStudio (version 1.1.463-^©^ 2009–2019 RStudio Inc., Boston, USA).

## Results

### Genetic associations with coeliac disease phenotypes

Of the previously identified 94 coeliac disease-associated variants, 39 were typed to sufficient extent and thus were selected for the study. When comparing coeliac disease patients to controls, ten SNPs were associated with specific phenotypes (Table 2, and Supplementary Table S2). Rs5979785 in the *TLR7*–*TLR8* region was associated with coeliac disease diagnosis ≤7 years of age and stratifying for gender revealed that this association only occurs in girls (Breslow–Day test, *P* < 0.001). This SNP was associated with the age of coeliac disease diagnosis in girls (OR = 0.11; 95% CI = 0.03–0.46) also in the case only analysis comparing coeliac disease patients with the given phenotype to those without (detailed results in Supplementary Table S3). As regards coeliac disease diagnosis above 7 years of age in the case–control analysis, rs653178 (at *SH2B3/ATXN2*), rs13010713 (at *ITGA4/UBE2E3)*, rs13151961 (at *IL2/IL21)*, rs11712165 (at *CD80*) and rs10936599 (at *MYNN*) showed an association. Except for rs10936599, the same variants were also associated with an intermediate HLA risk. Moreover, rs653178, rs13010713 and rs13151961 showed an association with the presence of gastrointestinal symptoms and with the presence of more severe mucosal damage (TVA/SVA) at diagnosis. In addition, rs653178 was also associated with extraintestinal manifestation as well as coeliac disease associated conditions in general and more specifically with AITD and T1D.

**Table 2:** Associations of genotyped coeliac disease risk variants to different phenotypes in the Finnish case–control material

Phenotypes	*N*	Marker	Gene	Chr	A1/A2	MAF all	MAF cases	MAF controls	OR (95% CI)	*P*_EMP2_
Gender, age										
*Female, CeD diagnosed* ≤ *7 years old*	30	rs5979785	TLR7/TLR8	X	G/A	0.25	0.03	0.24	0.11 (0.03–0.47)	0.015*
Age										
*CeD diagnosed* > *7 years old*	573	rs653178	SH2B3/ATXN2	12	C/T	0.42	0.47	0.41	1.28 (1.12–1.46)	0.015
		rs13010713	ITGA4/UBE2E3	2	T/C	0.50	0.45	0.51	0.78 (0.69–0.90)	0.015
		rs13151961	IL2/IL21	4	C/T	0.11	0.08	0.12	0.66 (0.52–0.84)	0.027
		rs11712165	CD80	3	G/T	0.40	0.45	0.39	1.26 (1.10–1.44)	0.031
		rs10936599	MYNN	3	A/G	0.27	0.31	0.26	1.27 (1.10–1.47)	0.046
CeD related symptoms in childhood	273	rs13098911	CCR1/CCR3	3	T/C	0.14	0.21	0.14	1.58 (1.26–1.99)	0.003
Gastrointestinal symptoms	526	rs653178	SH2B3/ATXN2	12	C/T	0.42	0.47	0.41	1.29 (1.12–1.48)	0.012
		rs13010713	ITGA4/UBE2E3	2	T/C	0.50	0.45	0.51	0.79 (0.69–0.91)	0.038
		rs13151961	IL2/IL21	4	C/T	0.11	0.08	0.12	0.66 (0.51–0.84)	0.030
Malabsorption										
* All*	267	rs17810546	IL12A	3	G/A	0.11	0.15	0.1	1.57 (1.21–2.04)	0.024
		rs2327832	OLIG3/TNFAIP3	6	C/T	0.19	0.26	0.19	1.53 (1.24–1.89)	0.002
* Anaemia*	157	rs2327832	OLIG3/TNFAIP3	6	C/T	0.19	0.28	0.19	1.74 (1.34–2.25)	0.001
Extraintestinal manifestations										
* All*	263	rs653178	SH2B3/ATXN2	12	C/T	0.42	0.48	0.41	1.36 (1.13–1.63)	0.042
* Neurological disorders*	38	rs13098911	CCR1/CCR3	3	T/C	0.14	0.28	0.14	2.34 (1.41–3.91)	0.034
* Fractures*	70	rs10936599	MYNN	3	A/G	0.27	0.41	0.26	1.84 (1.30–2.60)	0.021
CeD-associated conditions										
* All*	123	rs653178	SH2B3/ATXN2	12	C/T	0.42	0.52	0.41	1.61 (1.24–2.09)	0.012
		rs2298428	UBE2L3/YDJC	22	T/C	0.29	0.39	0.29	1.60 (1.23–2.09)	0.019
* AITD*	100	rs653178	SH2B3/ATXN2	12	C/T	0.42	0.54	0.41	1.68 (1.26–2.24)	0.012
* T1D*	18	rs653178	SH2B3/ATXN2	12	C/T	0.42	0.69	0.41	3.32 (1.63–6.67)	0.027
Small bowel mucosal damage										
* TVA/SVA*	361	rs653178	SH2B3/ATXN2	12	C/T	0.42	0.48	0.41	1.35 (1.15–1.58)	0.012
		rs13010713	ITGA4/UBE2E3	2	T/C	0.50	0.42	0.51	0.70 (0.59–0.82)	2E-04
		rs13151961	IL2/IL21	4	C/T	0.11	0.07	0.12	0.57 (0.42–0.78)	0.012
		rs2327832	OLIG3/TNFAIP3	6	C/T		0.24	0.19	1.37 (1.13–1.66	0.051
* PVA*	185	rs13098911	CCR1/CCR3	3	T/C	0.14	0.21	0.14	1.59 (1.21–2.08)	0.030
Intermediate HLA risk^a^	517	rs653178	SH2B3/ATXN2	12	C/T	0.42	0.47	0.4	1.35 (1.16–1.58)	0.006
		rs13010713	ITGA4/UBE2E3	2	T/C	0.50	0.45	0.52	0.73 (0.63–0.86)	0.004
		rs13151961	IL2/IL21	4	C/T	0.11	0.08	0.12	0.63 (0.48–0.82)	0.029
		rs11712165	CD80	3	G/T	0.40	0.45	0.39	1.29 (1.11–1.51)	0.048
CeD-antibodies										
* Negative CeD-autoantibodies*	19	rs13098911	CCR1/CCR3	3	T/C	0.14	0.34	0.14	3.19 (1.62–6.28)	0.020

A total of 1817 controls were used in all the analysis. Associations that resisted the 10,000 permutation corrections for multiple testing (*P*_EMP2_ ≤ 0.05) are reported in the table along with the respective odds ratios (ORs), 95% confidence intervals (95% CI) and corrected *P* values (*P*_EMP2_)

*N* sample size, *Chr* chromosome, *A1/A2* Minor Allele_Major Allele, *MAF* Minor allele frequency, *AITD* autoimmune thyroidal disease, *TVA/SVA* total or subtotal villous atrophy, *PVA* partial villous atrophy, *CeD* coeliac disease

^a^Cases and controls categorised as low HLA risk were dropped out from the analysis, and HLA intermediate risk participants included. Logistic regression adjusted the effect of gender on SNPs. The Breslow–Day (BD) test was used examine the homogeneity of the ORs in each gender strata and a *P_BD_ value < 0.02 was considered statistically significant. All markers were in Hardy–Weinberg equilibrium in the controls (*P* > 1 × 10^−6^)

In order to address the association of all the 94 coeliac disease associated SNPs, we carried out imputation. Out of the 2442 subjects in our study, 299 individuals (285 cases and 14 controls) were excluded due to heterozygosity and missingness. Of the 94 coeliac disease SNPs selected, five variants were missing (rs859637, rs2327832, rs12928822, rs58911644, rs4819388) and one (rs12998748) was multiallelic in the Finnish reference panel, and thus dropped out of the analysis. All the remaining 88 coeliac disease variants were successfully imputed and tested with the phenotypes (detailed results in Supplementary Table S4).

According to the results exploiting the imputed SNPs, five of the genotyped SNPs (rs5979785, rs653178, rs13010713, rs13151961, rs17810546) remained associated with the same phenotypes as in the original analysis. Seven imputed SNPs (rs3184504 at *SH2B3/ATXN2*, rs76830965 at *IL12A*, rs7616215 at *CCR1*, rs17264332 at *OLIG3/TNFAIP3*, rs243323 at *SOCS1*, rs13132308 at *IL2/IL21* and rs990171 at *IL18R1*/*IL18RAP)*, revealed as associated (*P* ≤ 0.001, adjusted for sex) with distinct phenotypes (Table 3, and Supplementary Table S4). Of these, rs76830965 was associated with coeliac disease diagnosis ≤7 years of age, the presence of gastrointestinal symptoms and malabsorption, intermediate HLA risk and coeliac disease autoantibodies. Moreover, rs3184504 was associated with coeliac disease diagnosis >7 years of age, extraintestinal manifestations, coeliac disease associated condition in general and with T1D specifically and also with intermediate HLA risk. The rest five imputed SNPs were associated with only one phenotype. Since 47.8% of cases were dropped out from the association analysis with the imputed SNPs, we carried out the remaining analysis with the 39 genotyped SNPs.

**Table 3:** Associations of imputed 88 coeliac disease risk variants to different phenotypes in the Finnish case-control material. Variants with imputation data only are highlighted in bold

Phenotypes	*N*	Marker	Gene	Chr	A1/A2	MAF all	MAF cases	MAF controls	OR (95% CI)	Beta	*P*_adj_ value
Gender, age											
* Female, CeD diagnosed* ≤ *7 years old*	25	rs5979785	TLR7/TLR8	X	T/C	0.25	0.06	0.25	0.19 (0.06–0.62)	−5.17	3.8E−04
Age											
* CeD diagnosed* > *7 years old*	311	rs653178	SH2B3/ATXN2	12	T/C	0.42	0.49	0.40	1.43 (1.21–1.70)	0.35	4.7E−05
		**rs3184504**	SH2B3/ATXN2	12	C/T	0.41	0.48	0.39	1.40 (1.18–1.66)	0.33	1.5E−04
		r**s76830965**	IL12A	3	A/C	0.09	0.13	0.08	0.62 (0.47–0.80)	−0.49	6.1E−04
		rs13010713	ITGA4/UBE2E3	2	C/T	0.50	0.44	0.51	0.76 (0.64–0.90)	−0.28	0.001
CeD related symptoms in childhood	170	rs5979785	TLR7/TLR8	X	T/C	0.24	0.15	0.25	0.52 (0.38–0.71)	−0.87	3.2E−04
		**rs7616215**	CCR1	3	T/C	0.38	0.47	0.37	1.47 (1.17–1.83)	0.37	0.001
Gastrointestinal symptoms	288	**rs76830965**	IL12A	3	A/C	0.09	0.13	0.08	0.58 (0.45–0.76)	−0.55	1.5E−04
		rs653178	SH2B3/ATXN2	12	T/C	0.41	0.48	0.40	1.36 (1.14–1.62)	0.30	7.4E−04
Malabsorption											
* All*	151	**rs76830965**	IL12A	3	A/C	0.09	0.16	0.08	0.48 (0.34–0.67)	−0.75	4.7E−05
		rs17810546	IL12A	3	G/A	0.10	0.16	0.10	0.57 (0.41–0.79)	−0.56	0.001
* Anaemia*	96	**rs17264332**	OLIG3/TNFAIP3	6	G/A	0.19	0.29	0.18	0.56 (0.41–0.78)	−0.58	8.0E−04
Extraintestinal manifestations											
*All*	139	rs653178	SH2B3/ATXN2	12	T/C	0.41	0.53	0.40	1.64 (1.29–2.10)	0.49	8.0E−05
		**rs3184504**	SH2B3/ATXN2	12	C/T	0.40	0.51	0.39	1.59 (1.24–2.03)	0.45	2.7E−04
* Neurological disorders*	22	**rs243323**	SOCS1	16	G/A	0.30	0.09	0.30	4.36 (1.56–12.21)	1.45	0.001
CeD−associated conditions											
* All*	64	rs653178	SH2B3/ATXN2	12	T/C	0.41	0.58	0.40	2.04 (1.43–2.91)	0.71	9.1E−05
		**rs3184504**	SH2B3/ATXN2	12	C/T	0.40	0.55	0.39	1.86 (1.30–2.65)	0.61	0.001
* T1D*	12	**rs3184504**	SH2B3/ATXN2	12	C/T	0.40	0.79	0.39	5.86 (2.18–15.72)	1.74	8.3E−05
		rs653178	SH2B3/ATXN2	12	T/C	0.40	0.79	0.40	5.65 (2.10–15.16)	1.72	1.1E−04
Small bowel mucosal damage											
* TVA/SVA*	205	rs13010713	ITGA4/UBE2E3	2	C/T	0.50	0.42	0.51	0.72 (0.58–0.88)	−0.34	0.001
* PVA*	102	rs13151961	IL2/IL21	4	G/A	0.11	0.04	0.12	2.84 (1.45–5.59)	1.04	4.2E−04
		**rs13132308**	IL2/IL21	4	G/A	0.11	0.04	0.12	2.84 (1.45–5.59)	1.04	4.1E−04
Intermediate HLA risk^a^	275	rs653178	SH2B3/ATXN2	12	T/C	0.41	0.50	0.40	1.46 (1.22–1.75)	0.38	3.8E−05
		**rs3184504**	SH2B3/ATXN2	12	C/T	0.40	0.48	0.39	1.41 (1.18–1.69)	0.34	2.2E−04
		**rs76830965**	IL12A	3	A/C	0.09	0.14	0.08	0.55 (0.42–0.73)	−0.60	3.6E−05
		rs17810546	IL12A	3	G/A	0.11	0.15	0.10	0.62 (0.48–0.80)	−0.48	3.8E−04
		**rs990171**	IL18R1, IL18RAP	2	G/A	0.50	0.56	0.49	0.75 (0.63–0.90)	−0.30	0.001
CeD-antibodies											
* Positive CeD-autoantibodies*	156	**rs76830965**	IL12A	3	A/C	0.09	0.14	0.08	0.56 (0.39–0.78)	−0.61	0.001

A total of 1749 controls were used in all the analysis

*N* sample size, *Chr* chromosome, *A1/A2* Minor Allele_Major Allele, *MAF* minor allele frequency, *TVA/SVA* total or subtotal villous atrophy, *PVA* partial villous atrophy, *CeD* coeliac disease.

^a^Cases and controls categorised as low HLA risk were dropped out from the analysis, and HLA intermediate risk participants included. Imputation analysis was carried out by using a Finnish population-specific panel of 3775 high-coverage (25–30×) whole-genome sequences (SISu v3) under the genome build version 38 (GRCh38/hg38). Phenotype-association testing was performed by using post-imputation genotype probabilities by using logistic regression under an additive model with the SNPTEST v2.5 software [30]. A frequentist test with Newml method corrected for gender. The ChrX-specific SNPTEST stratify-on gender method was used for associations in the ChrX region. Associations that reached our 10,000 permutation threshold (*P* value ≤ 0.001) are reported in the table along with their respective odds ratios (ORs), 95% confidence intervals (95% CI), beta coefficient and adjusted *P* values (*P* adj value). All imputed variants had good imputation metrics (INFO score of ≥0.9) MAF > 0.01, and were in Hardy–Weinberg equilibrium in the controls (*P* > 1  × 10^−6^)

### Functional annotation and pathway enrichment analysis

Of the 10 phenotype-associated genotyped SNPs, four variants (rs11712165, rs10936599, rs2327832 and rs13098911) had strong regulatory function on transcription with a RegulomeDB score ≤ 3a, indicating that the variant likely lies within a potential functional region. Of these rs13098911 was enriched with proxies with the lowest scores (1a and 1f). Out of the 148 extracted proxies to the other phenotype-associated SNPs (rs653178, rs13010713, rs17810546 and rs2298428), 42 had a putative functional role in gene regulation (RegulomeDB score ≤ 3a) (Supplementary Table S5). Thereafter we searched for eQTL effects for both the queried SNPs or the proxies that were likely to regulate gene expression or to be deleterious. We identified several significant highly tissue-specific eQTL effects (FDR < 0.05) (Supplementary Table S6). The eQTL genes connected to each of the phenotypes by the associated SNPs or their proxies were then subjected to KEGG pathways and GO terms analysis.

The analysis of the *cis*-eQTL genes of individual phenotypes, revealed significant enrichment only in the case of coeliac disease symptoms in childhood, neurological disorders, PVA and negative coeliac disease antibodies (Supplementary Table S7). The four cis-eQTL genes (*CCR5*, *CCR3*, *CCR2*, *CXCR6*) were enriched (FDR ≤ 0.002) in chemokine signalling and cytokine-cytokine receptor interaction KEGG pathways. These genes are likely to be involved in modulation of chemokine and cellular defence responses, cell chemotaxis second messenger-mediated signalling and divalent inorganic cation homoeostasis based on the GO associated terms (FDR ≤ 0.0001). Coeliac disease diagnosis above 7 years of age, gastrointestinal symptoms, coeliac disease-associated conditions, EI manifestations and intermediate HLA risk were the phenotypes connected to a set of *trans-eQTL* genes. Among KEGG pathways, they were most significantly enriched (FDR ≤ 0.01) in the B cell receptor signalling, Pertussis, NOD-like receptor signalling and in MicroRNAs in cancer. They were enriched (FDR ≤ 2.2E-09) for biological processes GO terms mostly involved in adaptive immune response, T cell activation, response to interferon-gamma, leucocyte cell-cell adhesion, cytokine secretion, regulation of leucocyte activation and response to molecule of bacterial origin (Supplementary Table S7).

### Genetic risk score and polygenic risk score associations with coeliac phenotypes

In order to study the combined effect of the 39 SNPS on different phenotypes, we applied the wGRS39 tertiles (Supplementary Table S8). Patients at the highest wGRS39 tertiles had significantly higher risk for having coeliac disease-related symptoms during childhood (OR = 1.76, 95% CI = 1.12–2.77), a more severe small bowel mucosal damage (OR = 1.76, 95% CI = 1.08–2.90), malabsorption (OR = 1.62, 95% CI = 1.04–2.54) and anaemia (OR = 1.68, 95% CI = 1.03–2.78) (Table 4, and Supplementary Table S8).

**Table 4:** Association of weighted genetic risk score (wGRS) (in tertiles) with coeliac phenotypes at disease diagnosis in the Finnish coeliac population

	Coeliac disease-related symptoms during childhood	
wGRS39 tertiles^a^	OR [95% CI]	*P value*
*Low*	Reference	
*Medium*	1.27 [0.84–1.92]	0.258
*High*	1.76 [1.12–2.77]	**0.014**

	Total/Subtotal villous atrophy	
wGRS39 tertiles^a^	OR [95% CI]	*P value*
*Low*	Reference	
*Medium*	1.31 [0.85–2.03]	0.220
*High*	1.76 [1.08–2.90]	**0.024**

	Malabsorption	
wGRS39 tertiles^a^	OR [95% CI]	*P value*
*Low*	Reference	
*Medium*	1.13 [0.75–1.71]	0.563
*High*	1.62 [1.04–2.54]	**0.035**

	Anaemia	
wGRS39 tertiles^a^	OR [95% CI]	*P value*
*Low*	Reference	
*Medium*	0.92 [0.58–1.50]	0.742
*High*	1.68 [1.03–2.78]	**0.041**

*CI* confidence interval, *OR* odds ratio

^a^Coeliac phenotypes were categorised into low-, medium- and high-risk wGRS39 tertiles, according to the distribution of the average weighted risk alleles in the controls. Phenotypes grouped by wGRS39 tertiles with fewer than five events in the reference group were not tested

**Fig. 1:**
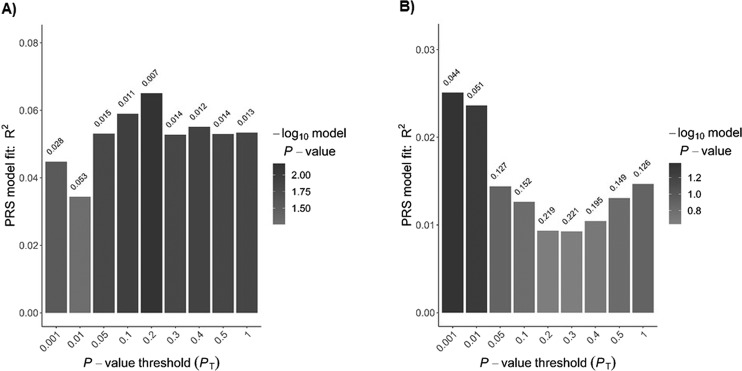
Polygenic risk score (PRS) predictions of the presence of dermatitis herpetiformis (**a**) and fractures (**b**) in coeliac patients at diagnosis. Values above each bar are unadjusted *P* values of phenotype from regression analysis

As regards the PRS analysis we found a significant effect of PRS on DH at *P* values threshold (*P*_T_ of 0.2, *R*^2^ = 0.06, *P* = 0.007, *P*_EMP2_ = 0.02) (Fig. 1a). Best fit PRS at *P*_T_ of 0.001 predicted the presence of fractures but did not persist permutation correction (*R*^2^ = 0.025, *P* = 0.043, *P*_EMP2_ = 0.12) (Fig. 1b, and Supplementary Table S9).

## Discussion

In this study, by using genotyped variants in our cohort, we identified a genotype-phenotype association for ten SNPs previously associated with coeliac disease susceptibility. In addition, our results demonstrate that combining 39 coeliac disease-associated genotyped SNPs into wGRS39 was more informative than a PRS to assess the genetic risk for distinct phenotypes of coeliac disease.

According to our results, rs5979785 located in the proximity of the *TLR7* and *TLR8* genes was associated with a decreased risk of diagnosis before 7 years of age in girls and notably this association was detected also in the case only comparison. Our RegulomeDB analysis did not retrieve rs5979785 as having regulatory function of transcription although it has previously been reported to decrease the *TLR8* expression in the blood [5]. TLR7 and TLR8 are both members of the toll-like receptor family and they detect distinct forms of viral nucleic acids and initiate antiviral responses [32]. As distinct viruses, including enterovirus has been implicated as a risk factor for coeliac disease [33, 34], additional studies on this loci in relation with viral infections and early coeliac disease onset are called for.

Our results also revealed an association of rs653178 at *SH2B3/ATXN2* with an increased risk to several other phenotypes, including concomitant T1D. Our findings are thus in accordance with previous studies where the 12q24/SH2B3 locus has been associated with both coeliac disease and T1D [8, 35, 36]. In these studies, rs3184504, a functional proxy in LD with rs653178 [37], has been indicated as the true SNP behind the association. Although rs3184504 was not included in our initial set of genotyped SNPs, we found it to be associated with concomitant T1D in the analysis carried out with the imputed genotypes.

In addition to having a likely regulatory function on transcription and an eQTL effect of the expression of SH2B3 along with many other genes in our analysis, rs3184504 SNP has been also associated with a higher expression of SH2B3 in the intestinal mucosal of patients with active coeliac disease [38], islet autoimmunity [39] and also implicated with bacterial infections [40], making the 12q24/SH2B3 locus associations in our cohort interesting for further research.

In pursue of clarifying the molecular mechanisms exerted by the susceptibility SNPs on the phenotypes, we identified that only three of our phenotype-associated SNPs had significant eQTL effects on regulating gene expression. This would suggest that the SNPs included in our analysis are mostly proxies and either situated in the proximity of the causative variant or further away. In the KEGG pathway and GO biological process analysis the phenotypes-associated rs13098911 SNP was identified as the only variant having *cis*-eQTL effects on a set of genes enriched in chemokine pathway. This finding most likely reflects the location of this variant in the chemokine gene cluster.

The enrichment results of the *trans*-eQTL genes identified largely identical pathways for most of our phenotypes. All these phenotypes were associated with rs653178 and its proxy rs3184504 revealed considerable effects on the expression of several distal genes. This pleiotropic effect of rs3184504 thus likely explains our finding of the pathway enrichment analysis of the *trans*-eQTL genes. Thus, further studies to infer the true phenotype-causal variants and the mechanisms that might mediate their effects on phenotypic variation are needed.

Regarding our weighted 39 SNP-based risk score model, the high-risk tertile in wGSR39 was associated with a higher risk of having coeliac disease symptoms in childhood, more severe small bowel mucosal damage, malabsorption and the occurrence of anemia. These phenotypes can be considered as signs of a more severe disease course [2, 41], thus raising the possibility that increased number of coeliac disease susceptibility SNPs might predispose to a more severe disease. In contrast, combining information from thousands of genomic variants into a PRS contributed to explain very little of the variance in phenotypes apart from DH. One major reason for this might be related to the fact that genetic loci not directly associated with disease status in large case–control GWAS may moderate the relationship between the coeliac polygenic burden and phenotypes [7, 10]. Thus, the main genetic contribution to phenotypic variations seems to derive from loci associated with the disease susceptibility.

A major strength of the current study is the clinically well-characterised large cohort of coeliac patients. In addition, a strength is the careful phenotyping of the patients, allowing us to investigate the association of different genotypes with various phenotypes. Moreover, the imputation analysis allowed us to explore the phenotype association of further 49 previously coeliac disease SNPs not typed in our cohort. The use of the most comprehensive available imputation panel in Finnish population increased our opportunities for identifying new coeliac disease SNPs associated with the phenotypes. Our highly conservative procedures in the imputation analysis diminished the risk of false positive associations. It must be noted though that 47.8% of coeliac disease patients dropped out from the analysis. Moreover, for distinct phenotypes, we had a fairly low number of patients which affected the statistical power in some cases. Moreover, we included in our GRS study only the 39 genotyped SNPs from independent coeliac disease risk loci, and not all the 94 previously associated with coeliac disease, and it might be the case that other coeliac susceptibility SNPs have a modulatory role on the phenotypes

We conclude that independent coeliac disease-susceptibility loci are associated with distinct coeliac disease phenotypes, suggesting that distinct SNPs might play a role in modulating the disease presentation in a yet to determined mechanism. Moreover, while PRS seems not to explain the variance in phenotypes, more severe coeliac disease phenotypes could possibly be contributed by higher amount of coeliac disease risk SNPs. Our GRS approach might thus be useful to identify patients at risk of developing a severe disease course unless identified and treated early. Further studies with a larger number of SNPs are called for in independent well-characterised patient cohorts to better understand how genetic variants contribute to the different coeliac disease phenotypes.

## Supplementary information

Supplementary Table 1

Supplementary Table 2

Supplementary Table 3

Supplementary Table 4

Supplementary Table 5

Supplementary Table 6

Supplementary Table 7

Supplementary Table 8

Supplementary Table 9
